# A quantitative risk assessment for human *Taenia solium* exposure from home slaughtered pigs in European countries

**DOI:** 10.1186/s13071-019-3320-3

**Published:** 2019-02-12

**Authors:** Marina Meester, Arno Swart, Huifang Deng, Annika van Roon, Chiara Trevisan, Pierre Dorny, Sarah Gabriël, Madalena Vieira-Pinto, Maria Vang Johansen, Joke van der Giessen

**Affiliations:** 1 National Institute for Public Health and the Environment (RIVM), Center for Infectious Disease Control, P.O. Box 1, 3720 BA Bilthoven, The Netherlands; 2 Department of Biomedical Sciences, Institute of Tropical Medicine, Nationalestraat 155, 2000 Antwerp, Belgium; 3 Department of Veterinary Public Health and Food Safety, Faculty of Veterinary Medicine, Ghent University, Salisburylaan 133, 9820 Merelbeke, Belgium; 4 Department of Veterinary Medicine, Universidade de Trás-os-Montes e Alto Douro, UTAD, Quinta de Prados, 5000-801 Vila Real, Portugal; 5 CECAV, Centro de Ciência Animale Veterinária, Universidade de Trás-os-Montes e Alto Douro, Quinta de Prados, 5000-801 Vila Real, Portugal; 6 Department of Veterinary and Animal Sciences, Faculty of Health and Medical Sciences, University of Copenhagen, Dyrlægevej 100, 1870 Frederiksberg C, Denmark

**Keywords:** *Taenia solium*, Cysticercosis, QMRA, Exposure, Meat inspection, Portion prevalence

## Abstract

**Background:**

*Taenia solium*, a zoonotic tapeworm, is responsible for about a third of all preventable epilepsy human cases in endemic regions. In Europe, adequate biosecurity of pig housing and meat inspection practices have decreased the incidence of *T. solium* taeniosis and cysticercosis. Pigs slaughtered at home may have been raised in suboptimal biosecurity conditions and slaughtered without meat inspection. As a result, consumption of undercooked pork from home slaughtered pigs could pose a risk for exposure to *T. solium*. The aim of this study was to quantify the risk of human *T. solium* exposure from meat of home slaughtered pigs, in comparison to controlled slaughtered pigs, in European countries. A quantitative microbial risk assessment model (QMRA) was developed and porcine cysticercosis prevalence data, the percentage of home slaughtered pigs, meat inspection sensitivity, the cyst distribution in pork and pork consumption in five European countries, Bulgaria, Germany, Poland, Romania and Spain, were included as variables in the model. This was combined with literature about cooking habits to estimate the number of infected pork portions eaten per year in a country.

**Results:**

The results of the model showed a 13.83 times higher prevalence of contaminated pork portions from home slaughtered pigs than controlled slaughtered pigs. This difference is brought about by the higher prevalence of cysticercosis in pigs that are home raised and slaughtered. Meat inspection did not affect the higher exposure from pork that is home slaughtered. Cooking meat effectively lowered the risk of exposure to *T. solium-*infected pork.

**Conclusions:**

This QMRA showed that there is still a risk of obtaining an infection with *T. solium* due to consumption of pork, especially when pigs are reared and slaughtered at home, using data of five European countries that reported porcine cysticercosis cases. We propose systematic reporting of cysticercosis cases in slaughterhouses, and in addition molecularly confirming suspected cases to gain more insight into the presence of *T. solium* in pigs and the risk for humans in Europe. When more data become available, this QMRA model could be used to evaluate human exposure to *T. solium* in Europe and beyond.

## Background

*Taenia solium* is a zoonotic tapeworm, with pigs as intermediate hosts and humans as definitive hosts. Pigs can become infected by ingestion of *T. solium* eggs. When eggs are ingested, oncospheres hatch from them, penetrate the intestinal walls and migrate towards the muscles. The oncospheres develop into *T. solium* cysticerci within 60 to 70 days [1]. Humans can become infected when pork with *T. solium* cysticerci is eaten raw or undercooked [2]. The adult tapeworm manifests in the human intestines, causing taeniosis. Human taeniosis is often undiagnosed, with mainly abdominal pain and bloating as reported symptoms [3].

Humans can obtain cysticercosis from direct contact with tapeworm carriers, contaminated food or water or through autoinfection or self-infection due to lack of sanitation [4]. Besides muscles, humane predilection sites are the eyes, subcutaneous tissues and brain. In contrast to human taeniosis, human cysticercosis may cause major health problems. Neurocysticercosis (NCC) is the most severe form of human cysticercosis, where cysticerci localize in the central nervous system. NCC is responsible for almost a third of all preventable epilepsy in endemic regions, mostly situated in low income countries [5].

The risk factors for human cysticercosis include poor personal hygiene, poor pig-raising practices [6], a lack of safe drinking water and sanitary latrines [7], consumption of infected, undercooked pork and poor knowledge about cysticerci in meat products [6, 8]. These conditions prevail in low income countries where pigs are raised and consumed, i.e. most countries in Latin America, sub-Saharan Africa and South and Southeast Asia [5]. In Europe, 4% of all pig holders raise 91% of all pigs [9]. These farms hold at least 200 pigs and have a biosecurity that is designed to minimize the transmission of pathogens like *T. solium*. Besides the structure and hygiene of European farms, meat inspection is obligatory at slaughterhouses in the European Union (EU), according to European Regulation 854/2004, chapter IV [10]. As a result, every pig carcass in the slaughterhouse is checked for cysticerci. Since almost no cases are reported in Europe [11], *T. solium* seems to be only a minor foodborne agent in Europe. Nevertheless, various recently published papers conclude differently [12–16]. A systematic review on the epidemiology of *T. solium* and *Taenia saginata* showed that one or more *T. solium* taeniosis cases were diagnosed in 4 out of 18 countries in western Europe. Human cysticercosis was even reported in all countries except Iceland. Most of these patients had visited endemic countries, which might explain the acquired infection, but there are also patients that had never left their country [13, 15]. Autochthonous cysticercosis cases could come from travellers with a taeniosis infection. But, this does not explain the porcine cysticercosis, that is notified in Austria, Bulgaria, Germany, Poland, Romania, Serbia and Spain, all between 1999 and 2015 [12, 13, 16].

Apparently, the conditions necessary for the transmission of *T. solium* between pigs and humans still persist in some European countries. *Taenia solium* transmission *via* the home slaughter of pigs was considered as a risk factor for the exposure to *T. solium* as a result of a questionnaire administered to members of the COST action TD1302, the European Network on Taeniosis/Cysticercosis (CYSTINET) that reported that home slaughter of pigs takes place in several countries, often without proper meat inspection [17].

The aim of this study was to analyse the risk of *T. solium* exposure from home slaughtered pigs, in comparison to controlled slaughtered pigs in European countries using a quantitative microbial risk assessment model (QMRA) that addresses the chain from production to consumption of pork.

## Methods

**Fig. 1:**
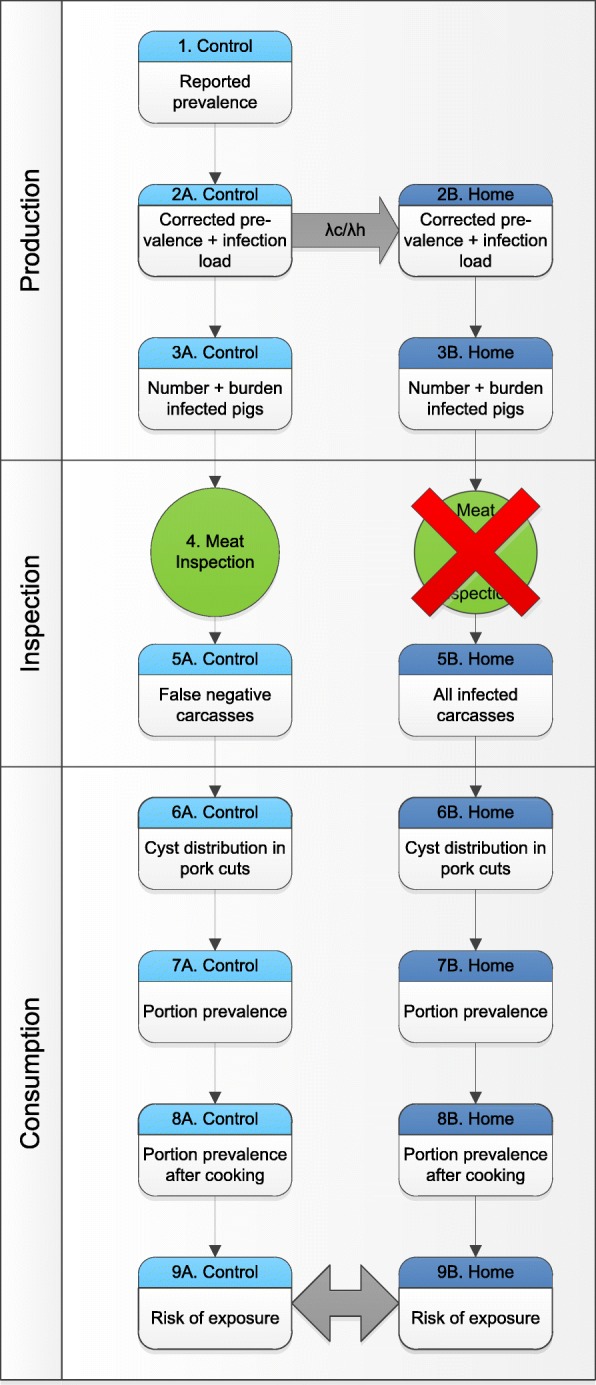
Conceptual risk chain for *T. solium* exposure

**Fig. 2:**
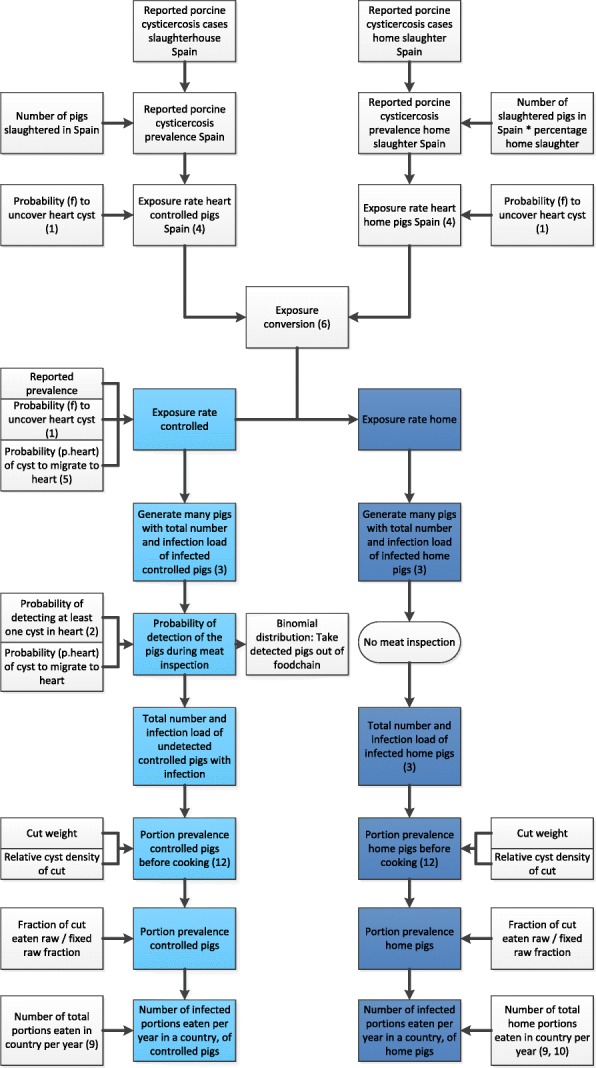
Model layout (formula numbers in parentheses)

We developed a QMRA model that followed the steps from porcine cysticercosis prevalence up until exposure of humans to infected pork portions. First, a general model description of the steps is given in Fig. 1. Secondly, the data sources and the calculations necessary to assess the risk of exposure per country are described in detail. The calculation steps are visualized in Fig. 2.

### Model description

The model was divided in three subsections: production, inspection and consumption. All steps were made at country level. The following steps were included (Fig. 1): (1) The model sets off with the reported prevalence in pigs. (2A) With calculations to determine the exposure rate and sensitivity of meat inspection, the adjusted prevalence and infection load of the porcine cysticercosis cases were defined. (2B) The adjusted prevalence and infection load of home slaughtered pigs was obtained using prevalence data of a country (Spain) where pork of home slaughtered pigs is inspected. (3) National data of the number of pigs slaughtered in slaughterhouses and outside slaughterhouses were multiplied by the prevalences of porcine cysticercosis to calculate the number of infected pigs for both controlled conditions (control) and home slaughtered conditions (home) apart. (4) Meat inspection, including test sensitivity was included in the ‘controlled’ branch of the model. For the ‘home’ branch, a comparison was made between meat and no meat inspection. (5) All carcasses which tested false negative were not withdrawn from the food chain and passed on to the section consumption. (6) With the aid of the infection load of the carcasses and the cyst distribution in pork cuts, the probability of a cysticercus to enter a cut was predicted (by “cut” we denote an anatomical part of the pig, such as “heart”, “loin”, etc.). (7) The weight of the cuts and a standard portion size were obtained to calculate the cyst distribution of the portions. By taking into account the total number of portions eaten in a country in a year, the portion prevalence and total number of infected portions could be obtained. (8) A subdivision between portions cooked and portions eaten raw was estimated. The portions consumed well-cooked were assigned zero risk, to calculate the final portion prevalence after cooking and thus to calculate (9) The risk of exposure.

### Data sources and calculations

#### Test sensitivity meat inspection

Official European examination of swine carcasses is described in chapter IV of the European Regulation 854/2004 [10]. This regulation lists all organs and muscles that need to be visually inspected. Regarding *T. solium*, the following organs need to be visually inspected: the tongue, diaphragm, pericardium and heart. Before 2013, the heart had to be incised lengthwise once, in order to view the ventricles and septum of the heart. As only the heart was cut to detect cysticerci, we assumed that the cut in the heart was the basis of European meat inspection for *T. solium* when we analyzed the data from Europe.

The sensitivity of meat inspection depends on the pig’s infection load [18]. To model this, the probability (*f*) to uncover a single cyst in the heart was calculated (formula 1).

1 $$ f=\frac{\mathrm{Mean}\ \mathrm{heart}\ \mathrm{surface}\ \mathrm{revealed}\ \mathrm{by}\ \mathrm{meat}\ \mathrm{inspection}\ \left({cm}^2\right)}{\mathrm{Mean}\ \mathrm{heart}\ \mathrm{surface}\ \mathrm{revealed}\ \mathrm{by}\ \mathrm{total}\ \mathrm{slicing}\ \left({cm}^2\right)} $$

The surface revealed by meat inspection is the area that can be inspected after the lengthwise incision mentioned above. Total slicing is the golden (standard) method to find *T. solium* cysticerci. Organs and muscles are sliced in 0.5 cm thick slices so that all cysticerci are uncovered. As such, total slicing gives the largest possible area that can be checked for *T. solium* cysticerci. The surfaces of formula 1 are adopted from Boa et al. [19].

The probability to find at least one cyst in the heart during detection was obtained with formula 2. When the total number of cysticerci in the heart *(n*_heart_*)* increases, the detection probability follows.

2 $$ P\left(\mathrm{detect}>0\ \mathrm{cysticerci}\ \right|\mathrm{cysticerci}={n}_{\mathrm{heart}}\Big)=1-{\left(1-f\right)}^{n_{\mathrm{heart}}} $$

#### Exposure rate and infection load

The exposure of pigs to *T. solium* eggs depends on certain risk factors that differ between countries and regions. We supposed that pigs are exposed to the eggs, resulting in an exposure rate (*λ*_heart_), of eggs in the heart per lifetime. The probability of having an infection with *n*_heart_ cysticerci was described by a Poisson distribution (formula 3), which is used for events that happen at random with a constant rate to an individual, i.e. an animal [20]. A higher exposure to eggs leads to a higher probability and infection load. When the exposure rate of a country is known, the formula can be used to determine the number and load of infected pigs with cysticercosis in that country, adjusted for the meat inspection sensitivity.

3 $$ P\left(\mathrm{cysticerci}={n}_{\mathrm{heart}}\right)=\mathrm{Poisson}\left({\lambda}_{\mathrm{heart}}\right)=\frac{\lambda_{\mathrm{heart}}}{n_{\mathrm{heart}}!}{e}^{-{\lambda}_{\mathrm{heart}}} $$

Note that the exposure rate is the rate of exposure of the heart per lifetime, since this is the muscle that the prevalence is derived from. In the section “Cyst distribution and weight of pork cuts” scaling factors are introduced to derive infection loads in other muscles. When combining formulae (2) and (3), the following formula results:

4 $$ P\left(\mathrm{detect}\right)=1-{e}^{-f{\lambda}_{\mathrm{heart}}} $$

where *P*(detect) is the probability to find a positive pig, given a certain *f* and *λ*_heart_. This probability of finding a positive pig is analogous to the reported prevalence in European countries, as the sensitivity of meat inspection and the exposure rate lead to found cases in the slaughterhouse.

We entered *f* and the reported prevalences as *P*(detect) in formula 4, yielding *λ*_heart_ for each country. To derive *λ*_pig_, the exposure rate of the whole pig instead of the heart, the exposure rate was divided by the probability of a cyst to develop in the heart (*p*_heart_) (formula 5).

5 $$ {\lambda}_{\mathrm{pig}}=\frac{\lambda_{\mathrm{heart}}}{p_{\mathrm{heart}}} $$

A binomial distribution was used to find all infected and non-infected pigs in the model, with *n* the number of pigs and *P*(detect) (formula 4) the probability of detection.

#### Prevalence

The reported prevalences were acquired in three steps. Firstly, the number of porcine cysticercosis cases per country was adopted from two reviews about the epidemiology of *T. solium* and *T. saginata* [13, 16]. An additional literature search was done for European countries that were lacking from the reviews [21, 22]. Secondly, for all countries that reported an annual number of cases but no total number of tested pigs, the total number of pigs slaughtered in slaughterhouses was taken from Eurostat [23]. Thirdly, the annual number of cases was divided by the annual number of slaughtered pigs to generate a prevalence of reported cases. This is the controlled reported prevalence, because all reported cases were found in slaughterhouses [13, 16, 21, 22].

The adjusted number of infected pigs originating from controlled housing was divided by the total number of pigs assessed to obtain the adjusted prevalence in a controlled setting. By “adjusted prevalence” we mean the reported prevalence, adjusted for the sensitivity of meat inspection (formula 3).

Home slaughtered pigs are more likely reared in uncontrolled housing systems. This could imply that home slaughtered pigs have also had a higher exposure to *T. solium*. This assumption is supported by data from Spain, where home slaughtered animals are inspected according to the same method as regularly slaughtered animals. The reported prevalence in Spanish pigs under controlled conditions ranges between 0.02–0.03%, while amongst home slaughtered pigs a prevalence of 0.16–0.43% is reported (2011–2013) [13]. The ratio between controlled and home reported prevalence in Spain was used to calculate the home prevalence in other countries in our model.

Initially, the controlled and home reported prevalence of Spain were entered in formula 4, attaining two exposure rates, the controlled exposure rate called${\lambda}_{\mathrm{heart}}^c$ and the home exposure rate${\lambda}_{\mathrm{heart}}^h$ . These were divided by *p*_heart_ to obtain ${\lambda}_{\mathrm{pig}}^c$ and ${\lambda}_{\mathrm{pig}}^h$ (formula 5).

Formula 6 demonstrates the step to the exposure conversion, that was applied in the model for all countries to convert the adjusted controlled prevalence in the adjusted home prevalence.

6 $$ \mathrm{Exposure}\ \mathrm{conversion}=\frac{\lambda^c\ }{\lambda^h} $$

#### Number of slaughtered pigs

The database Eurostat records the annual number of slaughtered pigs per European country, as well as the number of pigs slaughtered at places other than the slaughterhouse [23, 24]. Slaughtering ‘outside the slaughterhouse’ was adopted as home slaughtering in our calculations. The yearly slaughter records taken into account are the same years for which the national number of porcine cysticercosis cases is known. The average of these years was used in the model to calculate an average prevalence.

#### Cyst distribution and weight of pork cuts

The distribution of *T. solium* cysticerci in pig carcasses is not homogeneous. The predilection sites described are for instance the pork shoulder, pork leg and psoas muscle [25]. To take into account the cyst distribution in the model, literature data were used. In a paper of Boa et al. [19] naturally infected pigs were slaughtered and in every half carcass the cysticerci per muscle group or organ were counted by the total slicing method. The average amount of cysticerci per cut was divided by the average total cysticerci of the 24 pigs. The mean percentage of total cysticerci in the cut was divided by the mean percentage of the weight of that cut to calculate the relative cyst density [19]. The relative cyst density is the probability of a cyst being present in a cut. The relative cyst density of the heart was used in formula 5 as *p*_heart_. Also the relative cyst density was used in a binomial function that is defined in the section “Cysticerci per consumed portion”.

The weight of the pork cuts was not available from literature. Only the weights relative to the average carcass weight were given (Mean Weight %) [19]. To obtain the actual cut weights in kilograms, literature about porcine brain weights of pigs in the same age class was used, since brain weight is a stable proxy for age [26]. This Weight_brain_ was taken to convert the Mean Weight% of cuts to Weight_cut_. This is shown in formula 7.

7 $$ {\mathrm{Weight}}_{\mathrm{cut}}=\frac{{\mathrm{Weight}}_{\mathrm{brain}}}{\mathrm{Mean}\ \mathrm{Weight}{\%}_{\mathrm{brain}}}\ast \mathrm{Mean}\ \mathrm{Weight}{\%}_{\mathrm{cut}} $$

The trunk muscles, *musculus psoas*, *musculus triceps brachii*, forelimb, abdominal muscles and hindlimb were not deducted from the brain weight, because those are only parts of the pork cuts loin, tenderloin, shoulder, foreleg, belly and ham, respectively. For these cuts we assumed a homogeneous distribution within the complete cut, so that the relative cyst distribution of the muscles described in Boa et al. [19] could be used for the entire pork cuts that we assessed. The weight of these cuts was collected from literature [27–30].

#### Cysticerci per consumed portion

A couple of steps were followed to determine how many cysticerci end up in the consumed portions of all pork cuts. First of all, the number of portions per cut was calculated. Therefore, the cut fraction and the total number of portions consumed in a country were determined with the following formulae:

8 $$ \mathrm{Cut}\ \mathrm{fraction}=\frac{{\mathrm{Weight}}_{\mathrm{cut}}}{{\mathrm{Weight}}_{\mathrm{carcass}}} $$

9 $$ \mathrm{Total}\ \mathrm{portions}=\mathrm{Population}\ \mathrm{size}\ast \frac{\mathrm{Pork}\ \mathrm{consumption}\ \left(\frac{kg}{inhab}/ yr\right)}{\mathrm{Portion}\ \mathrm{size}\ (g)}\ast 1000\ (g) $$

10 $$ \mathrm{Total}\ \mathrm{portions}\ \mathrm{home}\ \mathrm{slaughter}\mathrm{ed}\ \mathrm{pigs}=\mathrm{Total}\ \mathrm{portions}\ast \mathrm{Fraction}\ \mathrm{home}\ \mathrm{slaughter} $$

Using samples from a multinomial distribution with probabilities given by formula 8, and the number of trials by formula 9 (‘controlled’) or 10 (‘home’), a distribution of cuts compliant to formula 8 was generated. Secondly, a binomial function was used to calculate the number of cysticerci that end up in a certain cut. The number of trials of the binomial function is the number of cysticerci in the pigs, calculated in step 2 of the risk chain model. The probability of a cyst entering a cut is equal to the relative cyst density that was described before. Thirdly, the probability of a cyst in a cut being present in a portion from this cut is equal to the fraction portion (formula 11).

11 $$ \mathrm{Fraction}\ \mathrm{portion}=\frac{{\mathrm{Weight}}_{\mathrm{portion}}}{{\mathrm{Weight}}_{\mathrm{cut}}} $$

With this proportion as probability, and the cysticerci per cut as number of trials, a second binomial distribution provided the number of cysticerci in a portion. The abovementioned binomial distributions were applied to every portion that was annually eaten in a country, thus giving the total of infected portions. The total number of portions eaten from controlled pigs was derived from formula 9. This number was multiplied by the fraction home slaughtered pigs to obtain the total number of home slaughtered portions in a country (formula 10). The final outcome is the portion prevalence (formula 12).

12 $$ \mathrm{Portion}\ \mathrm{prevalence}\ \left(\%\right)=\frac{\mathrm{No}.\mathrm{of}\ \mathrm{infected}\ \mathrm{portions}}{\mathrm{Total}\ \mathrm{no}.\mathrm{of}\ \mathrm{portions}}\times 100\% $$

#### Cooking

As only raw or undercooked meat confers an actual risk to public health, cooking practices were appraised in the model. Two approaches were taken to differentiate between raw and cooked consumed portions. The first approach, cooking scenario 1, was an indicative estimation of raw consumption, with the aid of personal communication with traditional Dutch farmers who used to prepare home slaughtered pork, and some websites addressing pork cuts and cooking methods. In this approach, a specific estimation is given of what fraction of a cut is eaten raw. The second approach, cooking scenario 2, was based on three scenarios (2A, 2B and 2C): cooking 10, 50 and 90% of the cuts. In this approach a standard fraction of every cut is assumed eaten raw. We assumed perfect inactivation of cysticerci during cooking. So, only the fractions of the cuts estimated eaten raw have viable cysticerci according to the model.

After the cooking step, the final portion prevalence and total number of infected pork portions, originating from pigs raised under controlled housing and from home slaughtered pigs, could be determined for every country included in the model. Furthermore, the separate attribution of the cuts to the total portion prevalence was assessed.

#### Inclusion of variability

In several places in the model we employed variability distributions (e.g. the Poisson distribution for number of cysticerci). A single run of the model calculates a large number of pigs (one million), the result is a distribution over individual pigs, from which prevalences and numbers of infected portions could be calculated. Five hundred iterations of this model were performed and outputs stored. The means, 2.5% and 97.5% percentiles were calculated. These numbers represent variability in the output, not uncertainty. One should interpret these numbers as indications of natural variation that one may expect to see due to chance.

### Software

The quantitative risk assessment model was run in R v.3.4.3 [31], with data stored in Microsoft Excel 2010 spreadsheets.

## Results

Data were available for five countries on the prevalence of porcine cysticercosis and the number of home slaughtered pigs, namely Bulgaria, Germany, Poland, Romania and Spain. The results of these countries are presented henceforth.

### Test sensitivity of meat inspection

**Fig. 3:**
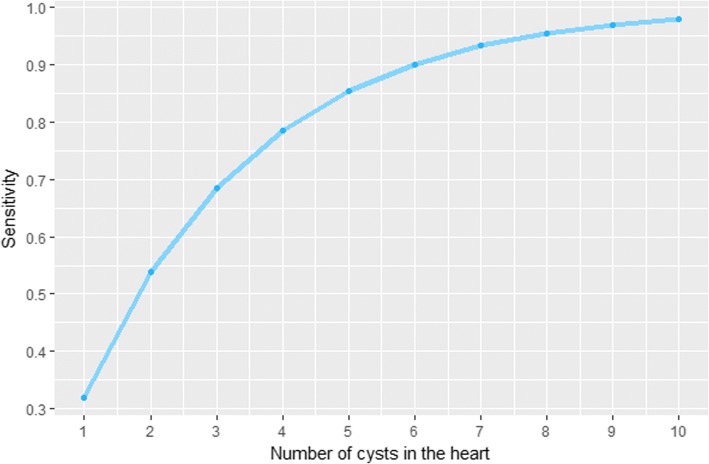
Sensitivity of meat inspection, dependent on cysticerci in the pigs’ heart

As mentioned in the methods section, European meat inspection does not reveal all present cysticerci in pig carcasses. According to Boa et al. [19], the lengthwise incision of the heart that is performed during meat inspection gives access to 136 cm^2^ of the heart. Total slicing reveals 425 cm^2^. The inspection proportion of the area is 32%. In other words, when cutting the heart, each heart cysticercus has a probability of *f* = 0.32 to be detected [19]. From formula 1, with *f* = 0.32, the relation between the infection load of the heart and the sensitivity of the current method of European examination of swine carcasses was obtained. Figure 3 demonstrates this relationship, showing that meat inspection sensitivity is very low when there are only a few cysticerci in the heart and nearly 100% when there are ten or more cysticerci in the heart.

### Exposure rate and infection load

**Fig. 4:**
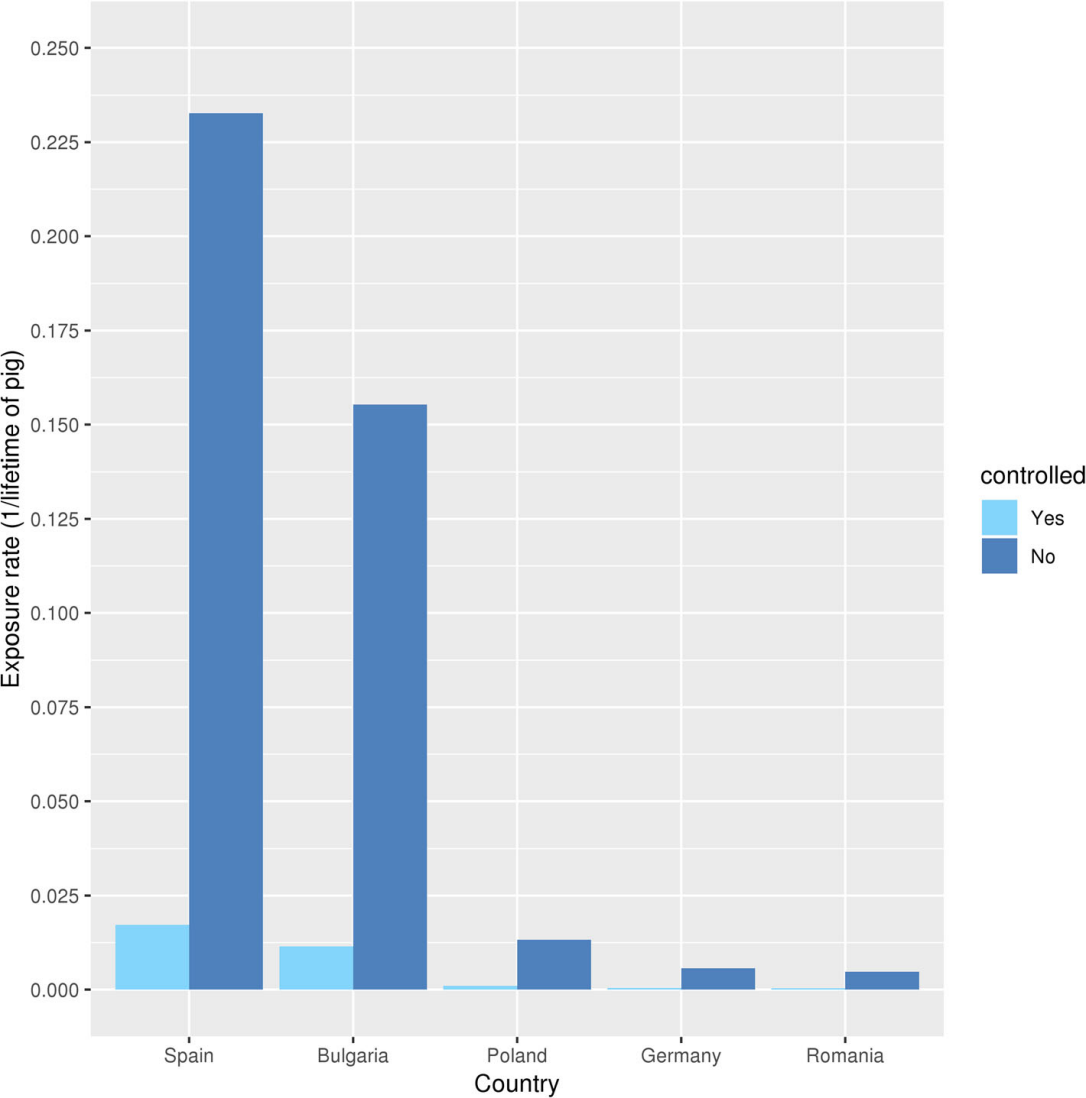
Exposure rate of pigs to *T. solium* eggs in a lifetime, by country and housing

With the aid of reported prevalences and the probability of finding a cysticercus in the heart, formula 3 led to the exposure rate of pig hearts to *T. solium* eggs (Fig. 4). The reported prevalences are given in Table 1.The calculated heart exposure rates for every country were corrected for the probability of any cysticercus to be located in the heart, *p*_heart_ = 3.6 × 10^-2^ [19] to obtain the ${\lambda}_{\mathrm{pig}}^c$. The calculated values of ${\lambda}_{\mathrm{pig}}^c$ are given in Table 1.

**Table 1:** Data inputs and parameters

Model part	Symbol: definition	Unit	Bulgaria	Germany	Poland	Romania	Spain	References
Test sensitivity meat inspection	*f*: probability of revealing a heart cysticercus	Proportion	3.2 × 10^-1^	3.2 × 10^-1^	3.2 × 10^-1^	3.2 × 10^-1^	3.2 × 10^-1^	Boa et al. [19]
Exposure rate	*Pheart*: probability of a cysticercus to enter the heart	Proportion	3.6 × 10^-2^	3.6 × 10^-2^	3.6 × 10^-2^	3.6 × 10^-2^	3.6 × 10^-2^	Boa et al. [19]
	λ^c^pig: exposure rate pigs in controlled housing	Rate	2.7 × 10^-2^	9.8 × 10^-4^	2.3 × 10^-3^	8.2 × 10^-4^	4.0 × 10^-2^	This study: Table 2
	λ^h^pig: exposure rate pigs in uncontrolled housing	Rate					5.4 × 10^-1^	This study: Table 2
Prevalence	*P*(detect): reported prevalence	Proportion	1.3 × 10^-4^	4.8 × 10^-6^	1.1 × 10^-5^	4.0 × 10^-6^	2.0 × 10^-4^	Laranjo-Gonzales et al. [13]; Trevisan et al. [16]; Oleleu et al. [22]; WAHIS interface OIE [21]
Slaughter data	Fraction home slaughter of pigs	Proportion	2.3 × 10^-1^	4.0 × 10^-3^	6.4 × 10^-2^	5.5 × 10^-1^	6.0 × 10^-4^	Eurostat [23, 24]; this study
Cysticerci distribution and weight pork cuts	Relative cyst density	Proportion	0.006–0.02	0.006–0.02	0.006–0.02	0.006–0.02	0.006–0.02	Boa et al. [ 19]
	Weight of cuts	kg	0.09–14.0	0.09–14.0	0.09–14.0	0.09–14.0	0.09–14.0	This study: Table 4
	Cut fraction	Fraction	2.2 × 10^-3^–3.3 × 10^-1^	2.2 × 10^-3^–3.3 × 10^-1^	2.2 × 10^-3^–3.3 × 10^-1^	2.2 × 10^-3^–3.3 × 10^-1^	2.2 × 10^-3^–3.3 × 10^-1^	This study: Table 4
	Fraction portion	Fraction	7.2 × 10^-3^–1	7.2 × 10^-3^–1	7.2 × 10^-3^–1	7.2 × 10^-3^–1	7.2 × 10^-3^–1	This study: Table 4
Cysticerci per portion	Average population	Inhabitants	7.5 × 10^6^	8.1 × 10^7^	3.8 × 10^7^	2.0 × 10^7^	4.7 × 10^7^	Eurostat [9]
	Pork consumption	kg/capita/yr	20.1	53.6	48.7	28.8	48.7	Faostat [37]
	Portion weight	g	100	100	100	100	100	This study
	Total portions	Portions/yr	1.5 × 10^9^	4.4 × 10^10^	1.9 × 10^10^	5.8 × 10^9^	2.3 × 10^10^	This study
Cooking	Scenario 1	Raw fraction	0–0.47	0–0.47	0–0.47	0–0.47	0–0.47	This study: Table 5
	Scenario 2a	Fraction	0.1	0.1	0.1	0.1	0.1	This study
	Scenario 2b	Fraction	0.5	0.5	0.5	0.5	0.5	This study
	Scenario 2c	Fraction	0.9	0.9	0.9	0.9	0.9	This study

### Prevalence

The reported prevalence of the countries included in the model is shown in Table 1. The adjusted prevalence of pigs that were raised uncontrolled and slaughtered at home was calculated *via* the exposure conversion (Table 2).

**Table 2:** Exposure conversion

Year	*λ*_*c*_	*λ*_*h*_	*λ*_*c*_ / *λ*_*h*_
2011	4.9 × 10^-4^	6.4 × 10^-3^	7.6 × 10^-2^
2012	3.8 × 10^-4^	5.1 × 10^-3^	7.4 × 10^-2^
2013	9.8 × 10^-4^	1.3 × 10^-2^	7.3 × 10^-2^
Average	6.1 × 10^-4^	8.3 × 10^-3^	7.4 × 10^-2^

**Fig. 5:**
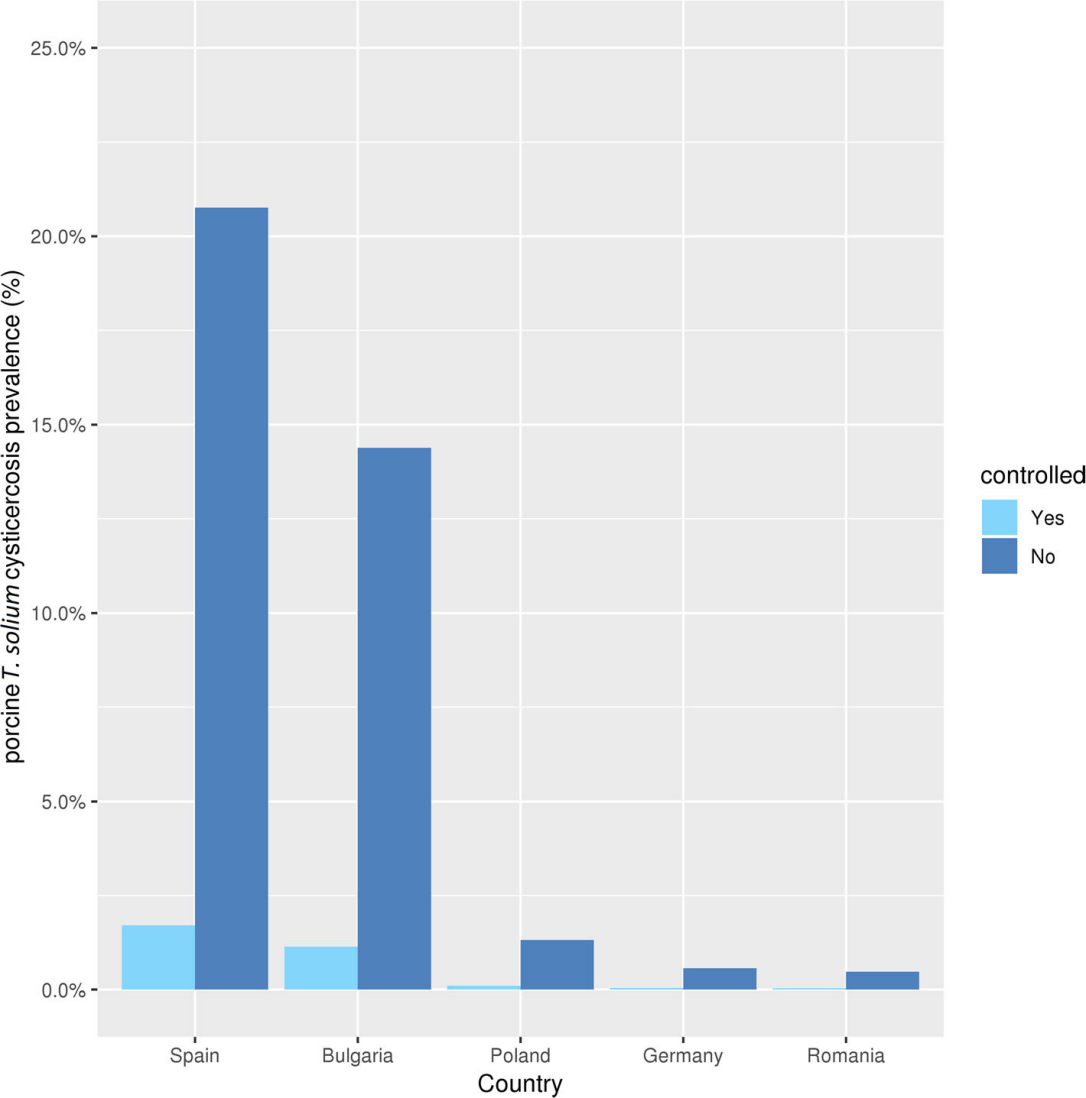
Adjusted *T. solium* prevalence of controlled and home slaughtered pigs, by country

In Table 3, column 3 shows that the calculated adjusted prevalence of pigs in controlled housing is approximately 86 times higher than the reported prevalence due to the low sensitivity of meat inspection, especially with a low infection load. The calculated adjusted prevalence of home slaughtered animals is another 12–14 times higher than the calculated controlled pig prevalence (Table 3, column 5). The highest prevalences are found in Spain and Bulgaria (Fig. 5).

**Table 3:** Prevalence of controlled and home slaughter

Country	Mean adjusted prevalence controlled (%) (95% V.I.)	Adjusted prevalence controlled/ reported prevalence controlled	Mean adjusted prevalence home (%) (95% V.I.)	Prevalence home/prevalence controlled
Bulgaria	1.128 (1.21–1.134)	86.75	14.387 (14.365–14.409)	12.76
Germany	4.145 × 10^-2^ (4.031–4.256 × 10^-2^)	86.36	5.663 × 10^-1^ (5.619–5.708 × 10^-1^)	13.66
Poland	9.668 × 10^-2^ (9.484–9.853 × 10^-2^)	87.89	1.316 (1.309–1.323)	13.61
Romania	3.53 × 10^-2^ (3.338–3.554 × 10^-2^)	86.32	4.721 × 10^-1^ (4.675–4.766 × 10^-1^)	13.67
Spain	1.684 (1.677–1.691)	84.21	20.76 (20.74–20.78)	12.33

*Abbreviation*: V.I., variability interval

### Slaughter data

The average fraction of home slaughter in the different countries is given in Table 1. Spain has the lowest fraction of home slaughter, namely 6.0 × 10^-4^. In Romania, more than half of the pigs are slaughtered outside slaughterhouses.

### Cysticerci distribution and weight of pork cuts

The relative cyst density and weight of cuts can be reviewed in Table 4. Pork organs or cuts that did not contain any cysticerci are not named as they are not relevant for the model. These are, for instance, the liver and kidneys [19]. The output of formula 6 is shown in column 4 of Table 2. The Weight_brain_ was set at 0.135 kg [26].

**Table 4:** *Taenia solium* cysticerci distribution and weight of pork cuts

Organ/Cut	Input data		Calculated data		
	Relative cyst density	Mean weight (%)	Weight (kg)	Cut fraction	Fraction portion
Brain	1.7 × 10^-2^	3.4 × 10^-1^	1.3 × 10^-1^	3.2 × 10^-3^	7.4 × 10^-1^
Head muscles	6.8 × 10^-2^	3.0	1.2	2.8 × 10^-2^	8.4 × 10^-2^
Internal masseter	1.6 × 10^-1^	2.3 × 10^-1^	9.1 × 10^-2^	2.2 × 10^-3^	1.0
External masseter	1.4 × 10^-1^	4.2 × 10^-1^	1.7 × 10^-1^	3.9 × 10^-3^	6.0 × 10^-1^
Tongue	5.9 × 10^-2^	1.1	4.4 × 10^-1^	1.0 × 10^-2^	2.3 × 10^-1^
Esophagus	5.5 × 10^-3^	2.4 × 10^-1^	9.5 × 10^-2^	2.2 × 10^-3^	1.0
Heart	3.6 × 10^-2^	8.1 × 10^-1^	3.2 × 10^-1^	7.6 × 10^-3^	3.1 × 10^-1^
Diaphragm	4.5 × 10^-2^	7.2 × 10^-1^	2.8 × 10^-1^	6.7 × 10^-3^	3.5 × 10^-1^
Tenderloin	2.0 × 10^-1^		5.0 × 10^-1^	1.2 × 10^-2^	2.0 × 10^-1^
Loin	2.0 × 10^-2^		1.4 × 10^1^	3.3 × 10^-1^	7.2 × 10^-3^
Shoulder	9.3 × 10^-2^		3.5	8.4 × 10^-2^	2.8 × 10^-2^
Foreleg	7.5 × 10^-2^		4.0	9.4 × 10^-2^	2.5 × 10^-2^
Belly	2.4 × 10^-2^		5.4	1.3 × 10^-1^	1.8 × 10^-2^
Ham	6.0 × 10^-2^		1.2 × 10^1^	2.9 × 10^-1^	8.2 × 10^-3^
Total	1		4.2 × 10^1^	1	

### Cysticerci per consumed portion

The cut fraction determined with formula 8 and the fraction portion with formula 11 are shown in the last two columns of Table 4. The portion weight and number of portions that are annually eaten in the five included countries are demonstrated in Table 1. The results from the binomial distributions used in this step, present the number of infected portions that consumers are actually exposed to, if all portions would be eaten raw.

**Fig. 6:**
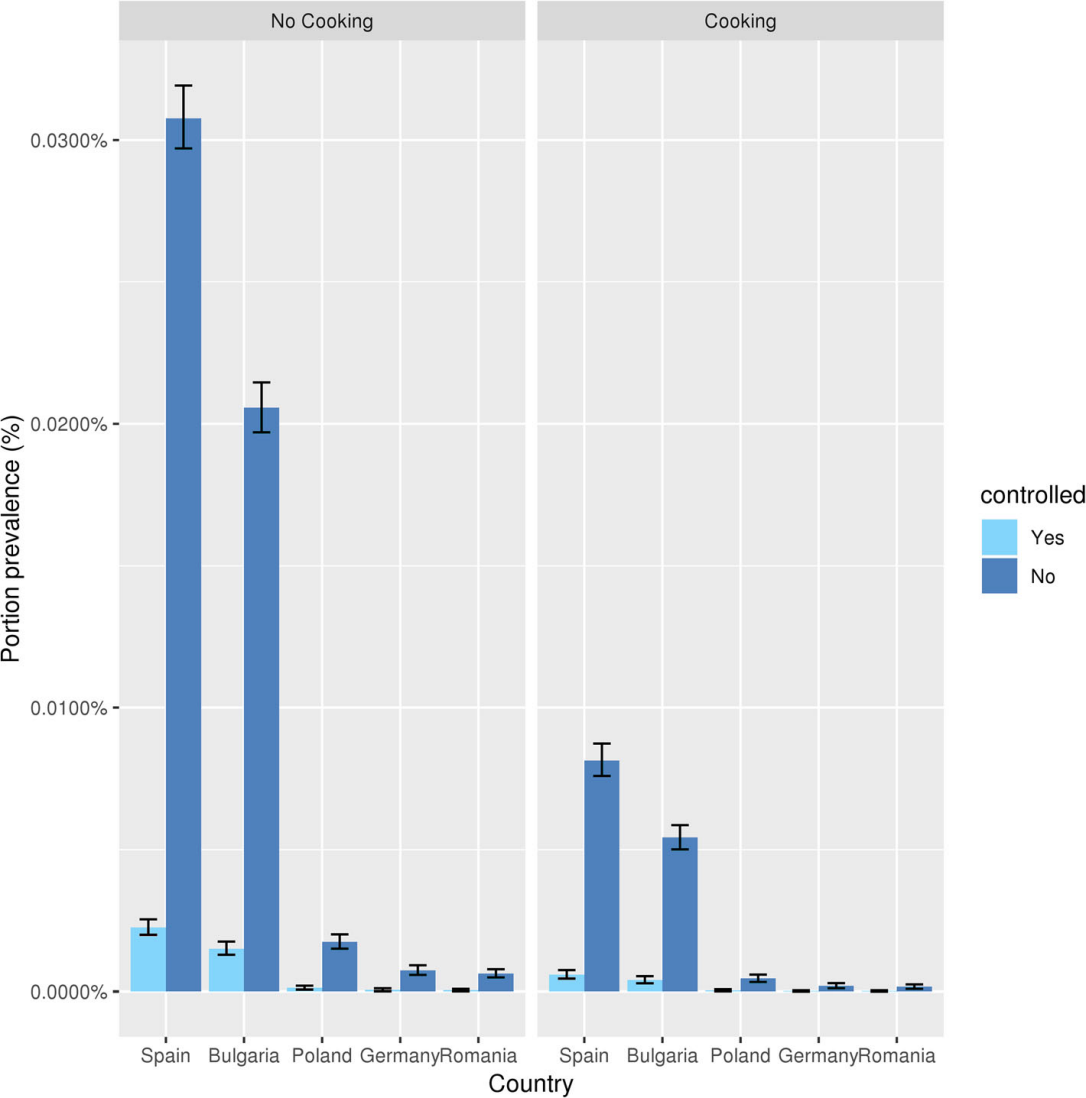
*Taenia solium* cysticerci infected pork portions prevalence by country and housing, before and after cooking compared

The portion prevalence is highest in Spain and Bulgaria, where 0.03% and 0.02% of the 100 g portions are infected, respectively, when pigs are slaughtered at home (Fig. 6; no cooking). The variability intervals (V.I.) are relatively small, which can be interpreted as little natural variation when prevalences are repeatedly calculated from hypothetical large samples of portions.

**Fig. 7:**
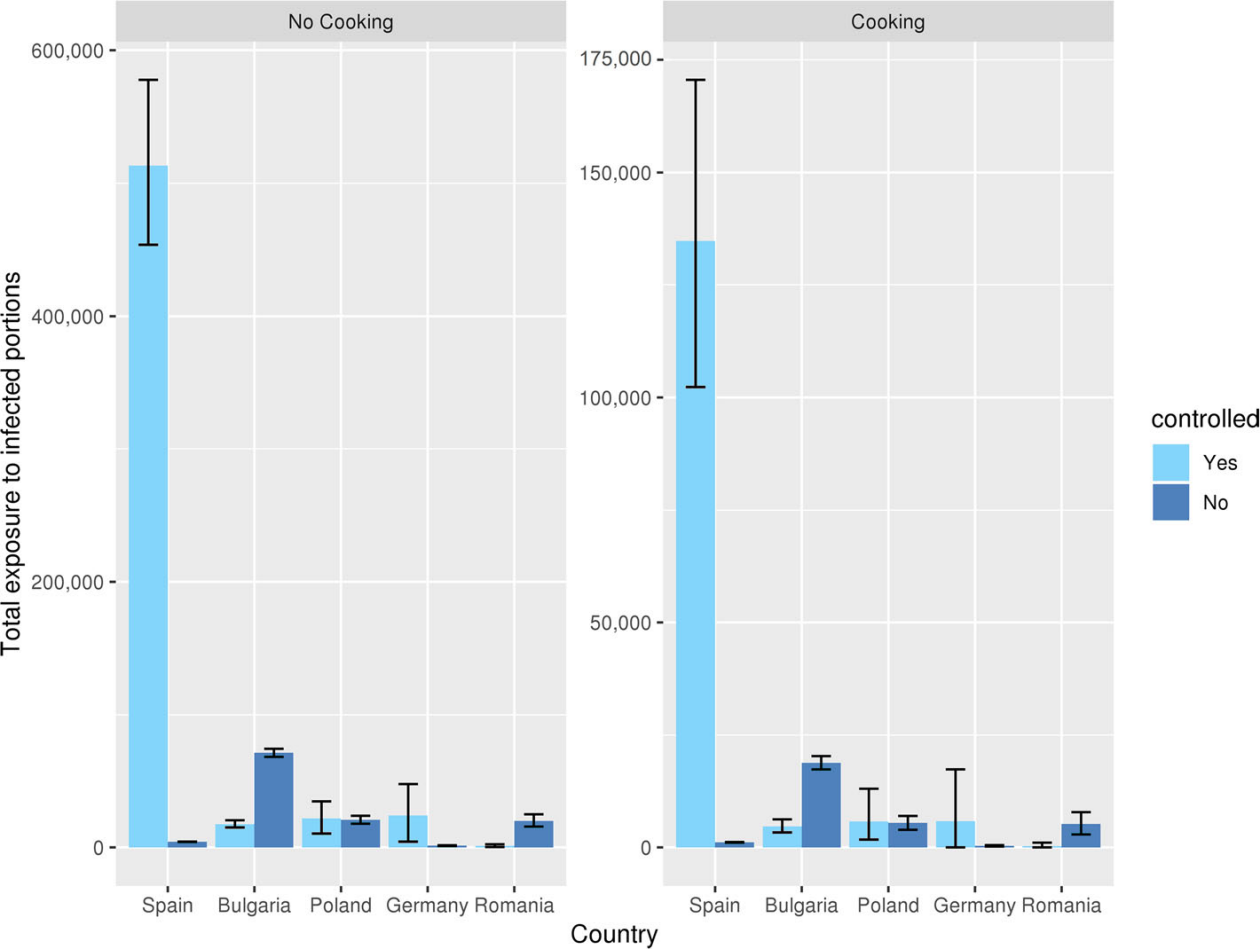
Total exposure to *T. solium* cysticerci infected pork portions by country and housing, before and after cooking scenario 1 compared

In Spain and Germany, the total number of infected portions (actual number of portions consumed times the portion prevalence of contamination) is higher under controlled conditions than when home slaughtered, while in Poland it is almost equal and in the other countries this is the other way around (Fig. 7; no cooking). Again, variability is limited, with some notable exceptions, namely Germany and Poland under controlled conditions. This means that estimates of numbers of contaminated portions are likely to give varying results over multiple (hypothetically comparable) surveillance results. The V.I.’s for Spain are large in an absolute sense, but not when viewed relative to the large absolute number of contaminated portions.

### Cooking

The cooking scenario 1 is described in Table 5. The *F*_*raw.prep*_ is the fraction that people are expected to eat raw. For example, the esophagus may be eaten raw when it is a component of ground pork. The *F*_*prep.eaten.raw*_ is the fraction of this ground pork that will be eaten raw instead of cooked. For the tenderloin, the whole cut is prepared undercooked, so the *F*_raw.prep_ is 1. Yet, the tenderloin is eaten medium/rare, so the whole cut has an *F*_prep.eaten.raw_ of 0.4. This gives a total raw fraction (*F*_raw.prep_ * *F*_prep.eaten.raw_) of the tenderloin of 0.4. Cooking scenario 2 is based on the second approach. As described in the methods, a fixed fraction of 0.1, 0.5 and 0.9 is considered to be eaten raw.

**Table 5:** Cooking scenario 1: Fraction of pork cuts eaten raw

Organ/Cut	Fraction of the cut prepared raw (*F*_raw.prep_)	Fraction of prepared raw, that is eaten raw (*F*_prep.eaten.raw_)	Total raw fraction (*F*_raw.prep_ * *F*_prep.eaten.raw_)	What raw products?
Brain	0	0	0	
Head muscles	0	0	0	
Internal masseter	0	0	0	
External masseter	0	0	0	
Tongue	0	0	0	
Esophagus	1	3.3 × 10^-1^	3.3 × 10^-1^	Ground pork in sausage
Heart	1	3.3 × 10^-1^	3.3 × 10^-1^	Ground pork in sausage
Diaphragm	1	3.3 × 10^-1^	3.3 × 10^-1^	Ground pork in sausage
Loin	1.7 × 10^-1^	3.6 × 10^-1^	5.9 × 10^-2^	Boneless top loin roast; sausage; bacon
Tenderloin	1	4.0 × 10^-1^	4.0 × 10^-1^	Baked medium/rare
Shoulder	2.5 × 10^-1^	3.3 × 10^-1^	8.3 × 10^-2^	Ground pork in sausage
Foreleg	0	0	0	
Belly	5.0 × 10^-2^	1	5.0 × 10^-2^	Bacon
Ham	5.0 × 10^-1^	9.4 × 10^-1^	4.7 × 10^-1^	Raw and cured ham; fricandeau: medium/rare

**Fig. 8:**
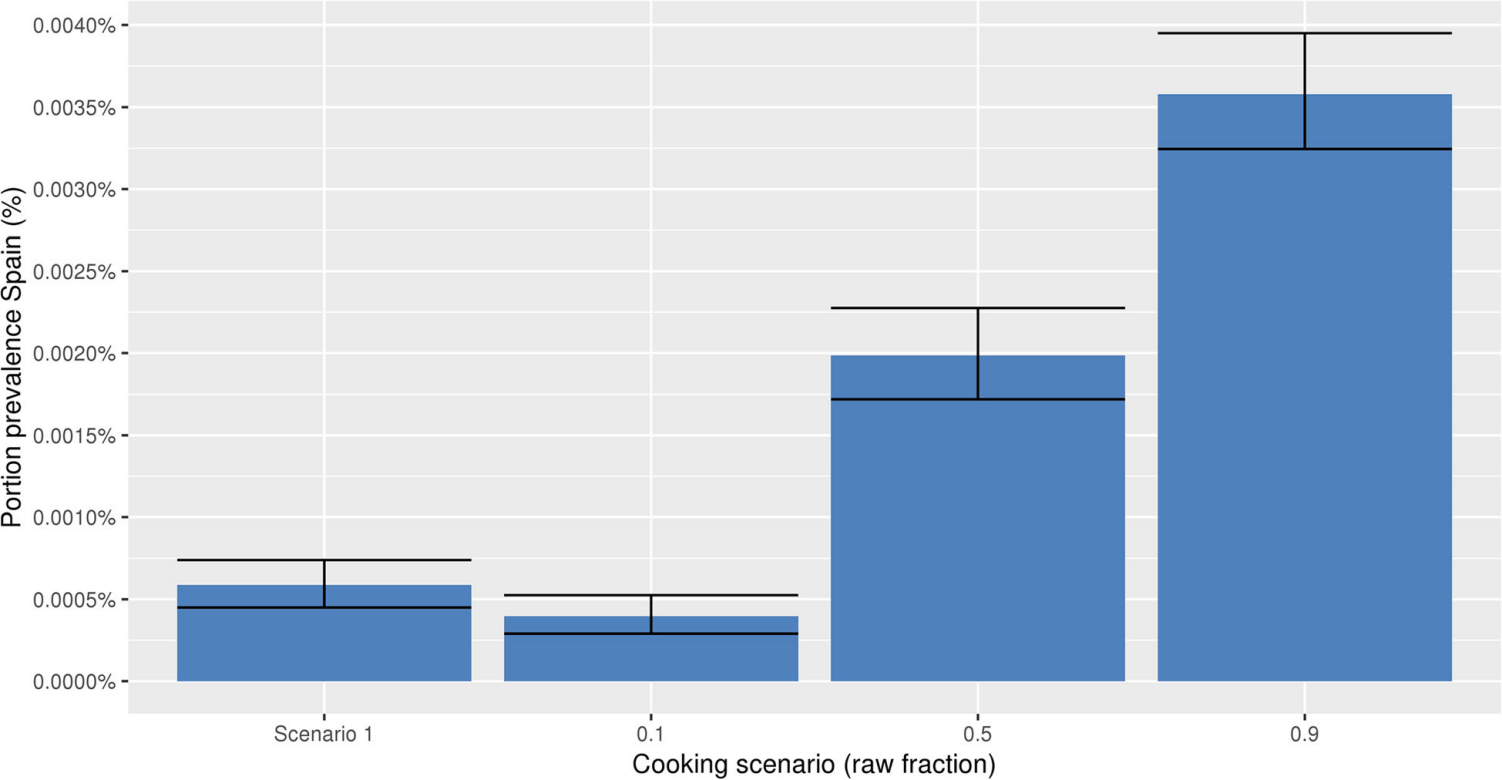
Prevalence of *T. solium* cysticerci infected pork portions after different cooking scenarios

The results of cooking pork are shown in Figs. 6, 7 and 8. In Fig. 7 the total exposure in all countries in a year is given, when the population of that country eats everything raw and when it cooks the portions as is estimated with scenario 1. These results can also be seen in Table 6. Cooking according to scenario 1 gives a 4 times lower total exposure of infected portions.

**Table 6:** Total exposure of the population to infected *T. solium* portions per country, before and after cooking the pork

Country	Before cooking		After cooking	
	Controlled mean (95% V.I.)	Home mean (95 V.I.)	Controlled mean (95% V.I.)	Home mean (95% V.I.)
Bulgaria	3.081 × 10^4^ (2.718 × 10^4^–3.448 × 10^4^)	1.251 × 10^5^ (1.211 × 10^5^–1.294 × 10^5^)	4.643 × 10^3^ (3.215 × 10^3^–6.244 × 10^3^)	1.884 × 10^4^ (1.746 × 10^4^–2.045 × 10^4^)
Germany	4.220 × 10^4^ (1.737 × 10^4^–6.947 × 10^4^)	2.325 × 10^3^ (1.953 × 10^3^–2.720 × 10^3^)	6.279 × 10^3^ (0–1.747 × 10^4^)	3.504 × 10^2^ (2.093 × 10^2^–4.883 × 10^2^)
Poland	3.861 × 10^4^ (2.511 × 10^4^–5.378 × 10^4^)	3.684 × 10^4^ (3.298 × 10^4^–4.098 × 10^4^)	5.576 × 10^3^ (0–1.214 × 10^4^)	5.473 × 10^3^ (3.914 × 10^3^–7.117 × 10^3^)
Romania	2.072 × 10^3^ (7.886 × 10^2^–3.680 × 10^3^)	3.524 × 10^4^ (2.831 × 10^4^–4.128 × 10^4^)	3.244 × 10^2^ (0–1.051 × 10^3^)	5.290 × 10^3^ (2.880 × 10^3^–8.000 × 10^3^)
Spain	8.996 × 10^5^ (8.163 × 10^5^–9.835 × 10^5^)	7.380 × 10^3^ (7.198 × 10^3^–7.565 × 10^3^)	1.335 × 10^5^ (1.023 × 10^5^–1.683 × 10^5^)	1.109 × 10^3^ (1.031 × 10^3^–1.186 × 10^3^)

*Abbreviation*: V.I., variability interval

The portion prevalence also decreases after cooking scenario 1 is applied. In Fig. 6 the portion prevalence before and after cooking is shown. A 50 times smaller portion prevalence remains after cooking. This is only shown for controlled pork, but for home slaughtered pigs the relative difference between before and after cooking is the same.

Scenario 2 is compared with scenario 1 in Fig. 8 for Spain. This figure demonstrates that if the raw fraction increases (e.g. 10 to 90%), more portions that are eaten contain viable *T. solium* cysticerci, according to the model. Cooking according to scenario 1 only leaves a higher portion prevalence than the in scenario 2 described raw fraction of 0.1. The figure only shows the results of controlled slaughter pigs in Spain since comparable results were obtained using the same scenarios for the other countries. The variability shown in Fig. 8 is limited, meaning that differences between these hypothetical cooking practices would in principle be observable with an appropriate population survey; natural variation alone will not make the scenarios indistinguishable.

**Fig. 9:**
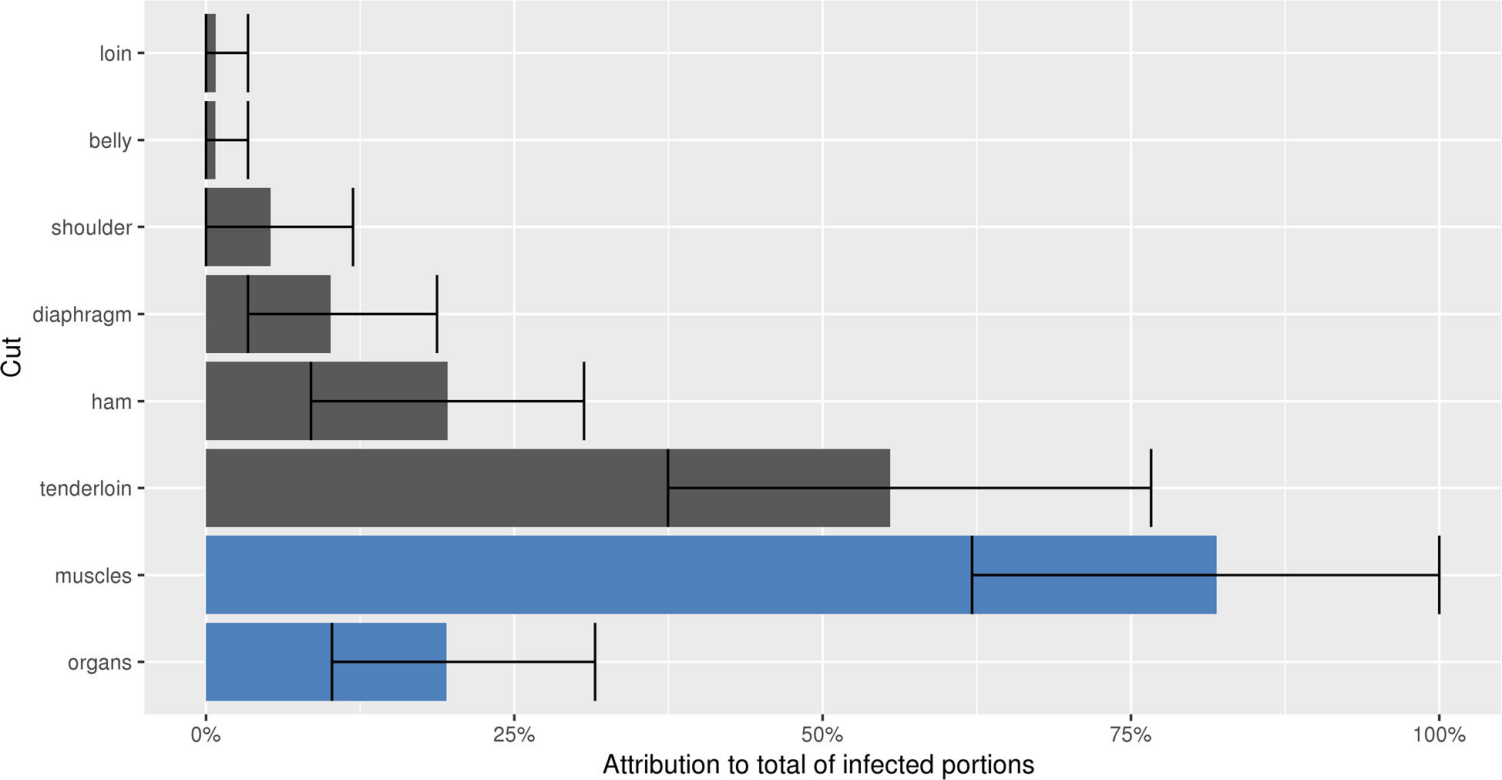
Attribution of cuts to the total of *T. solium* cysticerci infected pork portions

The attributions of the different cuts to the total exposure of consumers are shown in Fig. 9. The muscles are responsible for 80% (V.I. 62–100%) of the infected portions, and the organs for 20% (V.I. 10–30%). The organs that belong to this 20% are the esophagus, heart and diaphragm. The other organs are not eaten raw (Table 5). There is quite some overlap between the variability intervals, which means that due to chance alone the real ordering might be different. However, those cuts for which the intervals do not overlap will retain their relative ordering in the attribution.

## Discussion

We have built a quantitative microbiological risk assessment (QMRA) model for *T. solium* from pork production to consumption and implemented published data about the porcine cysticercosis prevalence, home slaughter numbers, the distribution of cysticerci in pork cuts and consumption quantities of pork. We present the results of the QMRA model for the risk of human exposure to *T. solium* due to consumption of pork in five European countries.

We demonstrated that the detection of *T. solium* cysticerci during meat inspection is dependent on the area of the body that is inspected and the infection load of the carcasses. The probability of finding an infected pig is low, since the reported *T. solium* prevalences in European countries are very low [13, 16] along with the sensitivity of meat inspection. This finding is in line with a study that evaluates meat inspection and other tests for the detection of cysticercosis [18]. We obtained the original data of that study to test our model (Dorny et al. [18]; unpublished data). The data consist of 65 pigs that were slaughtered, then inspected for *T. solium* according to the routine meat inspection protocol in that country and at last sliced to find all *T. solium* cysticerci [18]. Thirty-two pigs were infected with *T. solium*. We used our *p*_heart_ and formula 2 to determine which infected pigs would be found with meat inspection according to our model and compared it to the pigs that were actually found with meat inspection. With an arbitrary cut-off of 0.5 in our model, distinguishing between ‘detects’ and ‘non-detects’, our model had an error rate of 4/32. Just as meat inspection is a predictor of infection with a certain sensitivity and specificity, our model of meat inspection efficiency is also a predictor of “measured infection status”, with associated sensitivity and specificity. The sensitivity of our model on meat inspection is 75% and the specificity is 100%. The positive predictive value is 100%. The four pigs that were found infected during meat inspection, but not according to our model, could be predicted by our model per chance and due to the sharp cut-off of 0.5. Additionally, those four misdetects had fairly high numbers of cysticerci, casting doubt on the experimental outcome for those pigs. Furthermore, the meat inspection that was done in the study of Dorny et al. [18] included the heart and other organs like the masseter muscles while we only included the heart. Altogether, the predictive value of our model, regarding meat inspection, seems very high.

The exposure rates of home slaughter pigs are a factor 13.5 higher than those of controlled slaughter pigs as calculated from the exposure conversion that is derived from data of only one country, Spain. A home slaughter exposure rate based on prevalence data specific for the different countries would improve the outcomes of the model because uncontrolled housing of pigs or backyard pig keeping can exist over a wide range of husbandry practices. That affects the exposure of the pigs in that country. Although the exposure conversion is a substantial uncertainty in the model, the Spanish prevalence data did indicate that it is highly pertinent to take into account that home slaughtered pigs could have been subjected to a higher number of eggs in their lifetime than controlled slaughtered pigs. Furthermore, the Spanish data showed similar exposure conversions over the years, strengthening the idea that the estimate is robust.

By combining the sensitivity of meat inspection and the exposure rate we predicted the adjusted prevalence. That the calculated adjusted prevalence is about 86 times higher than the reported prevalence is not surprising, when we bear in mind the low sensitivity of meat inspection. Nonetheless, the prediction could be an overestimation because species misclassification might occur during inspection since the reported *Taenia* spp. cases were (except for Portugal) not confirmed by a diagnostic tool like polymerase chain reaction (PCR) [13, 16]. Other *Taenia* spp. for which pigs can serve as intermediate hosts are *T. hydatigena* and *T. asiatica*. However, there is no proof that *T. asiatica* is present in Europe, so we assume the contribution of this *Taenia* species to be negligible [32–34]. In addition, the porcine cysticercosis findings that are reported in slaughterhouses may be *T. hydatigena* cases, as this is a common parasite in Europe, especially in sheep raising areas. Nevertheless, the main predilection sites of *T. hydatigena* differs from *T. solium*, so a meat inspector should be able to distinguish between *T. hydatigena* and *T. solium* cases [35, 36].

We do realize that a bias is possible in the prevalence differences between countries. As the prevalence data depend on what is reported in the slaughterhouses, it is reasonable that the countries with more comprehensive reporting systems end up with the highest prevalence. As such, the fact that Spain and Bulgaria have the highest adjusted prevalence does not necessarily mean that their prevalence is indeed highest. In the ideal situation, the conversion from reported to adjusted prevalence could have been done separately for all countries. However, we only had these data from Spain and needed to assume the conversion is the same for the other countries. This also affects the remainder of the results. Because we used identical data for different countries, one can see patterns that are constant for the different countries, e.g. in Fig. 4. As a consequence, we addressed the results in general, instead of addressing it separately for each country.

In every country the home slaughter portion prevalence is a factor of 13 higher than the controlled slaughter portion prevalence. Regardless of this, the total number of infected portions was higher under controlled than home reared conditions in Germany and Spain. This can be explained by the small share of home slaughter in those countries, giving a very small number of total portions.

The portion size of 100 grams was chosen since the estimated consumption of pork meat is given in grams per capita per day that in the database of the FAO [37]. For the five countries in the model a consumption between 69 (Bulgaria) and 149 (Germany) grams per day has been reported. For the sake of clarity, the variety of portion sizes over the years and countries was not included in this risk assessment.

Since the portion prevalence is very low (shown in Fig. 6: the highest is 0.036%) the chance that someone gets exposed to more than one infected portion per year is very low. So we assume that every infected portion is eaten by someone else. For home slaughter though, this assumption might be wrong. If a family keeps some pigs for their own consumption and those pigs are all reared at the same place at the same time, all pigs might be infected and so the family has a much higher risk of exposure to *T. solium* cysticerci than the average. This illustrates that the portion prevalence for home slaughter is more complicated to translate to a quantitative risk on population level. The portion prevalence of home slaughtered pork was also assessed when meat inspection is done on home slaughtered carcasses. This is not shown in the results because the difference between the portion prevalences was negligible and remained as high as without meat inspection. We connect this to the low sensitivity of meat inspection. In a country with a higher exposure of the pigs, more pigs would be found infected in slaughterhouses (as the animal would have potentially multiple cysticerci in the heart, increasing the sensitivity of meat inspection), so meat inspection then would make a difference.

Fortunately, cooking of the pork portions goes along with a conversion in the number of infective portions. If the scenario that was presented in Table 5 is a good estimation of cooking practices, the risk is decreased by a factor 3 due to cooking. We have chosen scenarios where meat is either raw or perfectly cooked before being eaten, instead of a model where inactivation is a function of cooking time and temperature, as was done in a QMRA for another meat borne parasite *Trichinella* spp. [27]. Despite the fact that a publication about heat inactivation of *Taenia* cysticerci was available, the time to inactivation was not contemplated so we could not adopt these results for our model [38].

Estimating the fraction of a cut that is eaten raw is complex. Furthermore, the raw cuts are often dried, smoked or pickled with salt, and when a whole pig is slaughtered for one family a large quantity will be frozen. Freezing for four days at -5 °C, three days at -15 °C or one day at -24 °C effectively kills cysticerci [39]. Salt pickling lowers the viability of *Taenia* metacestodes due to changes in the osmotic potential, causing a membrane rupture [40]. The other preparation methods have not been evaluated as far as we know. Thus, the raw fraction of pork cuts eaten is a limitation of this study. Even with this considered, our model still has significant relevance, since raw meat consumption is common in European countries, although the consumption preferences depend on the cultural background and personal customs [41–44].

We identified a heterogeneous distribution of cysticerci in the pig carcasses in our model. The cyst density was used to calculate the share of each cut in the total of infected portions. The largest attribution after cooking comes from the tenderloin and ham. This might change when cooking is performed differently. For example the masseter muscles do not currently add to the risk because they are always eaten thoroughly cooked according to various sources [45–49]. They do, however, have a very high relative cyst density so if cooking habits change or a niche group prefers them raw, then the masseter would contribute to the risk. Another factor that could change the attributions of the cuts is the cysticerci viability in the meat. *Taenia* cysticerci can survive for three years after experimental infection [50]. In a study of pigs that were slaughtered 26 weeks post-infection, the mean total viability of cysticerci was 99% (SD ± 1) [51]. Pigs are often slaughtered around 20 weeks of age, so the assumption of 100% viability seems reasonable. Yet, these were experimentally infected pigs that received a single high dose of eggs, while naturally infected pigs are likely exposed to eggs all their lives. Furthermore, backyard pigs may be slaughtered at a later age, as they do not grow as efficiently as pigs in controlled housing. Studies with naturally infected pigs show that the viability fraction depends on the total infection load of the pigs (Dorny et al. [18] unpublished results) and varies over different cuts [19]. As we did not have enough data to include cysticerci viability in the model, we assumed that all cysticerci were viable.

In some countries the exposure from controlled housing is higher than from home slaughter (e.g. Spain, Germany), even though the portion prevalence of home slaughter is higher. This is due to much higher consumption frequencies of controlled produced pork. The average number of infected portions in the five countries assessed was 5.3 × 10^3^ according to the model.

To validate the model, the calculated portion prevalence must be compared with reported human taeniosis cases. However, many tapeworm cases will never be diagnosed due to the mild and vague symptoms [52]. Moreover, often the number of general taeniosis infections is reported, without specifying which *Taenia* spp. [13, 16]. Despite these concerns, in Poland from 2007 to 2009 a total of 278 human cases have been reported. One hundred and eighty cases were due to *T. saginata* and the other 98 cases were ‘other tapeworms’ (i.e. 35% of the total cases) [16]. If the other tapeworms were almost all *T. solium* cases it means there were on average around 33 cases per year. This would mean that from the annual 42644 infected portions, 0.08% result in reported infections. In Romania from 2007 to 2009, 1463 taeniosis cases have been reported. If in Romania 35% was also due to *T. solium*, around 170 cases per year would have been *T. solium* taeniosis cases [16]. This would imply that 0.8% of the total infected portions cause an infection. Although the difference between those countries a factor of 10, it is not impossible when we take into consideration the earlier described food customs in Romania and the percentage of home slaughter that is nine times higher in Romania than in Poland. As such, the number of people exposed as estimated by the QMRA model is reasonable, taking into account that the number of reported human cases is assumed an underestimation of the real cases.

## Conclusions

In conclusion, we developed a model to assess the relative exposure to *T. solium* in Europe, comparing pork originating from home slaughtered pigs with pork originating from controlled housing raised pigs. Our model takes into account different stages of the food chain, from the prevalence that starts at the pig farm to the portion prevalence that ends up on the consumer’s plate. This makes it possible to look at the effect of every step in the chain on the final exposure. The most important finding is that there is still a potential risk of a *T. solium* infection in Europe. This risk depends first on the reported porcine cases after meat inspection, which has a very low sensitivity, especially when pigs have a low infection load. Therefore, the adjusted prevalence of *T. solium* is much higher than reported, as we showed. Secondly, the portion prevalence of pork from home slaughter is 13.83 times higher than from controlled housed pigs. Thus, home slaughter is a very important risk factor for exposure to *T. solium*. Finally, exposure to *T. solium* depends on many factors and differs per country due to husbandry of pigs and cooking habits. The results of the model can be improved if more information about the prevalence among pigs (controlled and home slaughtered) and consumer behaviour regarding raw meat consumption is acquired. Therefore, it would be useful if European countries develop a better monitoring system for *T. solium* in pigs, preferably based on a more sensitive method instead of visual inspection [53] and molecular confirmation of suspected findings in the slaughterhouse. In addition, a comprehensive survey about raw meat consumption would reduce uncertainty in the estimates on the raw consumed portions and give a better perception of cultural differences (e.g. following the methodology of [54]). When these factors become better known, the QMRA model could support the assessment of human exposure to *T. solium*, both in and outside Europe.

## Abbreviations

F: Fraction
kg: Kilogram
NCC: Neurocysticercosis
QMRA: Quantitative microbial risk assessment
SD: Standard deviation
V.I.: Variability interval
